# “A Sane Island in an Ocean of Madness”: A Case of Alternative Organisational Ethics Through Post-Growth Values

**DOI:** 10.1007/s10551-024-05921-7

**Published:** 2025-01-09

**Authors:** Ben Robra, Alex Pazaitis, Arnaud Levy

**Affiliations:** 1 The Business School, University of Queensland, Brisbane, Australia; 2 P2P Lab, Ioannina, Greece; 3 Ragnar Nurkse Department of Innovation and Governance, Tallinn University of Technology (TalTech), Tallinn, Estonia; 4 Post-Growth Innovation Lab, University of Vigo, Pontevedra, Spain; 5 BUT Métiers du Multimédia et de l’Internet, Université Bordeaux Montaigne, Pessac, France; 6 noesya, Paris, France

**Keywords:** Post-growth, Organisational values, Autopoiesis

## Abstract

Unprecedented runaway climate change and ecological degradation is argued to be caused by the dominant capitalist mode of production’s reliance on endless economic growth and capital accumulation. Businesses and organisations are expected to act in an ecologically and socially ethical way to help avert the crisis. Yet, there has arguably been little progress in this direction. The conventional ethical frameworks are generally subsumed under capitalism’s reliance on growth that effectively delegate business ethics to a peripheral and, often, contradictory pursuit, insufficient to influence ecologically and socially sustainable business conduct. We therefore explore an alternative approach by operationalising business ethics through organisational values from a post-growth perspective. By analysing the case of a social cooperative, we highlight how post-growth organisational values emerge through the organisation’s history, the members’ experience, and active contrasting to the dominant capitalist value systems. We contribute to business ethics scholarship by highlighting the potential of post-growth organisational ethics and values in creating contrasts to the dominant capitalist values. Our research further contributes to sustainability scholarship, particularly post-growth perspectives, by highlighting that organising through post-growth values in contrast to the dominant economic system is not only possible, but essential to achieve sustainability. Ultimately, our research illustrates the need for political engagement in upholding organisational ethics, in the face of the ecological crisis.

## Introduction

Climate change and ecological degradation is having unprecedented effects on the planet’s ecosystems (Hoekstra & Wiedmann, 2014; Richardson et al., 2023). To avert potential ecological collapse, society must mitigate these changes (Hickel, 2020a; Kallis, 2018). Arguably, the endless and systemic pursuit of economic growth driven by capital accumulation is the main cause of climate change and ecological degradation as it requires constant expansions in material and energy use (Foster et al., 2010; Saito, 2017). The concepts of post-growth and degrowth^1^ have come to the forefront in challenging the dominant view of continuous growth as an inherently good, desired, and quasi-natural pursuit (Buch-Hansen, 2018; Schmelzer et al., 2022).

Post-growth scholarship argues that for a sustainable future, society’s material and energy consumption must be reduced on an absolute global scale through systemic changes that disentangle the economy from constant reliance on growth (Hickel, 2020b). Post-growth aims for a sustainable society in which overall economic activity decreases while well-being increases (Schneider et al., 2010). In this direction, post-growth scholarship ranges from questioning and relinquishing the aim of economic growth, to overcoming the capitalist social formation altogether (Akbulut, 2021; Schmelzer et al., 2022).

A tentative post-growth transition evidently entails profound implications for businesses and other forms of economic organisation^2^ (Shrivastava, 2015). Alignment with post-growth principles would arguably entail economic organisations to potentially forgo or downplay profit-maximisation and growth (Robra et al. 2020; Nesterova, 2020). Yet, organisational aspects have received relatively little attention in post-growth scholarship (Hankammer et al., 2021). Consequently, there is a shortage in feasible documentation of organisational practices informed by a post-growth perspective. The tentative role of organisations in enabling a post-growth society and the organisational forms best suitable for such a society has not been systematically explored. Likewise, engagement of organisation and business studies with post-growth remain scant and the view that businesses need to grow and maximise profits largely remains unchallenged (Banerjee et al., 2021).

Simultaneously, an ever-increasing call from academics, public policy influencers and laypeople alike for more ethical business conduct, especially in response to climate action, has, arguably, had limited impact. When it comes to the core organisational functions and practices, economic incentives and stimuli appear to dominate business decisions (Yazdani & Murad, 2015). Responsible conduct in response to societal and ecological challenges seems to be rather delegated to a peripheral aspect, subsumed under the profit maximisation imperative. Therefore, to examine whether and how organisations may align with post-growth principles and become equipped for a tentative transition, first we need to gain a deeper understanding of how business ethics emerge amidst our rapidly changing and uncertain economic and political context.

To do so, we engage with the concept of organisational values, as an operational interpretation of organisational ethics. Organisational values can be seen as an implicit basis for organisational decision-making and modus operandi (see Schnebel, 2000; Pruzan, 2001; Argandoña, 2003; Besio & Pronzini, 2014). By interpreting ethics through organisational values we may elicit a more nuanced understanding of the conditions under which organisations define their conduct. Organisational values do not emerge in a vacuum. Businesses operate within a system in which capitalism dominates. In this environment, organisations are compelled to grow and maximise profits; meaning ethical conduct needs to grapple with the imperatives of the dominant economic system. Particularly when it comes to prioritising values such as social justice or ecological responsibility that are alternative or even contradictory to the capitalist economic logic, it is vital to understand how such values emerge in organisations and formulate their ethical compass. Our main research inquiry is therefore how post-growth ideas can formulate the basis for an alternative organisational ethics. We address this question through a case study, by exploring the emergence of post-growth organisational values and identifying their sources and influence.

In this direction, our inquiry focuses on ‘value systems’ (Harvie & Milburn, 2010), seen as the basis for an organisation’s ethical framework constructed around a certain set of values. We propose a tentative theoretical framework to bridge two hitherto largely unrelated fields of inquiry, namely, organisational values and post-growth. Our theoretical lens borrows from Luhmann’s (2012) social system theory, on the one hand, and Marxist understandings of dominant political economic structures and resulting value systems, on the other (see Harvie & Milburn, 2010). Specifically, social systems theory allows us to examine organisational value systems in their system environment (Besio & Pronzini, 2014), as we elaborate below.

Drawing from social systems theory we adhere to a specific interpretation of how business ethics define right or wrong behaviour, whereby “[…] instead of making direct moral judgments, [ethics] reflects on values or on the correctness of specific moral approaches" (Besio & Pronzini, 2014, p. 290). These specific moral approaches depend on a system’s understanding of its system environment. This means that an organisation’s value system may be conditioned, but *not necessarily determined,* by its system environment. Through our Marxist view on political economic structures, we take the position that economic values in society are predominantly aligned with capitalist values. This means that for the majority of organisations, their system environments are dominated by capitalism and its value systems. Yet, it also means that organisational value systems in opposition to society’s dominant capitalist values can emerge. We posit post-growth values as alternative and/or contradictory to these dominant economic structures and logics.

The paper is structured as follows. Section “Theoretical Background” provides the theoretical background of the study, building on business ethics, organisational values, and value systems literature. In Sect. “Organising around Post-Growth Value Systems: A Tentative Analytical Framework”, we present our tentative analytical framework to explore organisational values from a post-growth perspective. Section “Methods” describes our case study approach. We present the studied organisation and our findings in Sect. “Case study”. In Sect. “Discussion” we discuss our findings with relevance to post-growth and business ethics scholarship alike. We briefly conclude in Sect. Concluding Remarks.

## Theoretical Background

### The Business of Ethics

Over the past four decades, a range of topics such as Corporate Social Responsibility (CSR) (Ehrnström-Fuentes & Böhm, 2022; Joyner & Payne, 2002) or Corporate Social Performance and Innovation (Wagner, 2010) have been adopted by a broad spectrum of organisations adhering to public demand for more ethical corporate action in response to environmental and social challenges. CSR measures in particular have become common practice to address social as well as environmental challenges (Blowfield & Murray, 2011; Málovics et al., 2008). Through the use of CSR, businesses generally seek to ensure their social licence to operate (Banerjee, 2008; Hilson, 2012). The general notion of these measures is to create so-called win–win situations in which a business activity not only creates economic benefits for the business but also social and/or environmental benefits (Carroll & Shabana, 2010; Porter & Kramer, 2006; Prieto-Carrón et al., 2006). Porter and Kramer (2006) in particular have put forward the notion that businesses should adopt measures such as CSR to capitalise on these win–win situations and gain competitive advantages. Arguably, the wide acceptance and adoption of CSR and other similar strategies by the business community can be explained by such measures fitting the business case and continued profit-making (Málovics et al., 2008).

An increasing body of literature questions the feasibility of CSR’s notion to continue the pursuit of profit maximisation while simultaneously protecting the environment and doing well for society (Banerjee, 2008; Dyllick & Hockerts, 2002; Heikkurinen & Bonnedahl, 2013; Prieto-Carrón et al., 2006; Yazdani & Murad, 2015). In the absence of formally enforced regulations, CSR and similar strategies have limited impact on the core economic activities responsible for climate change. They are merely a substitute form of self-regulation, entrusted to corporations’ ethical or moral tenets (Besio & Pronzini, 2014; Utting, 2005).

Meanwhile, the increasing scholarly engagement with business ethics has had limited success in translating moral or descriptive principles into business norms and practices, with the overruling motive of businesses remaining a purely profit-oriented one (Yazdani & Murad, 2015). Largely, the examination of business ethics has been focusing on the relationship between ethical conduct and financial performance (e.g., Waddock & Graves, 1997; Chun et al., 2013 in Yazdani & Murad, 2015). This treatment further reinforces the dominant perception that the sole purpose of organisations is ever-increasing profitability, with business ethics being merely another means to pursue it. In other words, the ethical business compass to act on issues such as climate change is often subjugated to the economic logic, i.e., climate action makes sense insofar it supports a for-profit purpose or at least does not undermine it (Besio & Pronzini, 2014).

Yazdani and Murad (2015, p. 400) call for a renewed take on business ethics, following a shift from ethics treated in terms of duties or work objectives, towards values that form “the core and whole of a person and an organisation” (see also Brady & Hart, 2007). There is a case to be made regarding organisational ethics formulated through axiological questions of what we should value as meaningful or important. Schnebel (2000, p. 80) argues that “[v]alues are the key factors in identifying the motivating aspects of decisions after the decision-making process in business, [containing] everything that can define, influence or shape the style of management and business execution of a corporation”. Similarly, Padaki (2000) defines organisational values as the organisation’s core convictions that become enduring practices over time. Organisational values can thus be seen as an (implicit) base for an organisation’s modus operandi and ethics (see Schnebel, 2000; Pruzan, 2001; Argandoña, 2003; Besio & Pronzini, 2014). Hence, to contemplate whether there is any place for ethics in business “in the face of corporate profit maximization orientation, global consumerist culture and unleashed capitalism” (Yazdani & Murad, 2015, p. 403) we need to delve into organisational values.

### Research on Organisational Values

The inquiry into organisational values emerged in the 1980s as a key facet of the organisational culture literature (Agle & Caldwell, 1999; Allaire & Firsirotu, 1984; Hofstede, 1980; Wiener, 1988). The concept of organisational culture is often said to be undefined or only broadly defined in the literature, in the sense that “the symbolic concept of culture provides a roof for a broad assortment of views about organizations” (Allaire & Firsirotu, 1984, p. 209). Similarly, and despite much research attention, unclarity and ambiguity of the term ‘organisational value’ in the literature persists (Agle & Caldwell, 1999; Argandoña, 2003).

Scholarly engagement with organisational values varies depending on how one interprets the term value(s). Various definitions have emerged in the organisational context, depending on the intended formulation or measurement (Wiener, 1988). To some extent, there is consensus on an understanding of organisational values as enduring forms of beliefs affecting behaviour in response to certain social expectations (see Rokeach, 1973; Wiener, 1988). The effect of the said beliefs becomes more evident when they are shared and, in varied degrees, become established, internalised or normatively guide behaviour and preferences among different modes of conduct or states of affairs related to the organisation.

Two core debates within organisational values literature concern the locus and sources of values. On the former, a key question is whether organisations, in an anthropomorphic way, can hold values themselves, as some scholars argue (see e.g., Agle & Caldwell, 1999; Pruzan, 2001), whereas others (see e.g., Hofstede, 1980; Wiener, 1988; Padaki, 2000) understand organisational values as a sum or cluster of shared values solely among an organisation's members. Concerning the sources of values, it is debated whether organisational values emerge on the micro level, i.e. within the organisation mainly through its members, or the macro level, i.e. the organisation’s societal setting and its values, in which it is situated and continues to reproduce (Padaki, 2000).

The above debates illustrate that interpretations of value and culture not only have a significant role in defining organisational values, but also the organisation itself. Hence, ontological and epistemological considerations on organisations play a key role in research on organisational values. Vincent and Wapshott (2014) suggest a starting point for any research on organisations is the ontological question of what the researcher(s) perceive an organisation to be. Likewise, an interpretive approach to the concepts of both value (singular) and values (plural) within a certain context can help us unravel their role in- and relation to the organisation and organising. An additional lens for interpreting organisational values depends not only on the definition of the organisation, as an entity, but also its processes and practices within society, as we elaborate in the following section.

### Organisational Value Systems

Many scholars speak of value systems as integral parts of organisational culture. Hofstede (1980, p. 24) defines organisational culture as “a system of collectively held values”. Similarly, Wiener (1988, p. 535) views values as part of the organisational culture and defines an organisational value system as follows:

“When a number of key or pivotal values concerning organization-related behaviors and state-of-affairs are shared—across units and levels—by members of an organization, a central value system is said to exist.”

Again, a clear emphasis here is on the organisation’s members’ views (see Sect. “Research on organisational values”). Building on the work of McMurtry (1998), Graeber (2001), and De Angelis (2007), Harvie and Milburn (2010, pp. 632–633) attribute value systems with a more pivotal role, that of defining an ethical framework for organisations, or rather, for organising as a process and practice:

“When we talk about values (plural)—as in ‘family values’, ‘Christian values’, ‘values of solidarity and mutual aid’ or ‘aesthetic values’—we are talking about practices, actions or relationships, the process of valuing. The idea of values refers to that which people hold dear, esteem or cherish; a value system refers to ethical framework constructed around a set of values. [...] Even when the cherished object is just that, an object, we are still talking about a mode of human behaviour, an action, the action of cherishing. More commonly when we think about values or value systems we are referring to relationships amongst humans.”

In this view, value systems are relational and refer to the ways human beings qualify certain forms of relating to each other over others. In turn, value(s) are not only based on evaluations of modes of conduct, but also modes of acting and relating. A value system emerges through clusters of modes affirmed as ‘good’ and other modes repudiated as ‘bad’ formulating a way of “thinking and acting in the world” (McMurtry, 1998, p. 7).

Hence, organisational value systems lean on ethical frameworks, within and beyond the organisation, i.e. organisational values formulate a basis upon which both individual and organisational actions are interpreted within the broader social whole, in which the organisation operates. Organisational value systems define the organisational perception of value (singular), as that, which is valued as important. Value is understood here not as an outcome of constituent processes but as a process per se, through which actions and relations acquire meaning, within a broader social whole (Pazaitis et al. 2022 cf. Graeber, 2001). Value systems, in this sense, are a form of collective agreement on what it means to act ethically, be it an individual or an organisation. Organisational values are conditioned by societal values, the latter providing the broader social whole within which good and bad practices are defined. As people draw meaning for their actions from these definitions, and act upon them, individual values largely become a reflection of the societal value system, as they articulate and reproduce it (De Angelis, 2007). Business ethics is, then, reflected on- and by organisational value systems that operate as conceptual grids, through which organisational actions and decisions are morally classified.

## Organising around Post-Growth Value Systems: A Tentative Analytical Framework

By engaging with organisational values, we aim to understand whether and how organisations may act upon ethical interpretations stemming from post-growth values. To do so, we need an analytical framework that can help us examine the processes through which organisational value systems emerge within a certain social context, and, in turn, reproduce themselves through the organisation. As a starting point, we employ Luhmann’s (2018) outlook on organisations as self-referential social systems that observe their system environment. We posit that organisations (re)produce their value systems through engagement with society and its respective value systems. However, as we approach this engagement, we take inspiration from Schecter (2019) to depart from Luhmann’s interpretation of society, towards a Marxian perspective that views capitalist values as dominating the majority of society’s values systems. In turn, we interpret post-growth organisational values as the basis for an alternative ethical framework emerging in contrast to these dominant capitalist norms.

### Emerging Organisational Value Systems

Luhmann (2018) interprets organisations as autopoietic social systems. Though a comprehensive overview of Luhmann’s theory would exceed the confines of this research, we need to engage with the main concepts from which our analysis departs. Most importantly, the ideas of autopoiesis and self-reference, which we try to explain below. Maturana and Varela (1980) first coined the term autopoiesis to describe the capacity of living systems to reproduce themselves, while reproducing their constituent components and conditions of reproduction. Luhmann (2012) abstracts and applies this concept to social systems, which he conceptualised as operationally closed, in the sense that social systems draw a distinction to their system environment and operate based on their own internal logic. Through this distinction, social systems interpret and understand the world (themselves and their environment), not in direct interaction with their environment, but through self-referential observations reflecting upon their own constituent operations.

A system’s self-referential logic, which becomes its autopoiesis, is reproduced and built through communication (Seidl & Becker, 2006). Communication can either be accepted or rejected, depending on how a system understands it in self-reference (Luhmann, 2012). Communication builds on previous communication, while a system only reproduces if its communication leads to further communication. Organisations as social systems reproduce through a particular kind of communication, decision communication (Luhmann, 2018). Organisations continuously communicate decisions, whereby decisions are based on previously communicated decisions, in other words these decisions become decision premises (Seidl, 2018). An organisation’s autopoiesis is structured by its decision premises, which define the organisation’s interaction with its system environment. However, an organisation is also influenced by its system environment and might adapt to it according to its own internal logic. Hence, even though the organisation as a social system is self-referential, it emerges in a wider societal setting.

Luhmann’s theory of organisations as social systems is very useful to examine how organisation’s align with certain values and ethical interpretations in their conduct, focusing on their internal operations, while reflecting on the broader societal context. However, to employ the social systems’ perspective we first need to address two points of tension between Luhmann’s theory and our conceptualisation of organisational value systems.

The first tension relates to the role of values in organisations. Values, within social systems theory, are considered as the basis for another form of communication, value communication (Baraldi et al., 2021; von Groddeck, 2011a). Luhmann (2008) laments that values are so abstract that they lack the ability to steer any action effectively, as it depends on how one understands and interprets a seemingly common value. Von Groddeck (2011a, p. 73) counters Luhmann’s view as it “emphasises that values cannot resolve conflicts or provide orientation in complex situations, but that is exactly the hope of business ethics researchers or managers who try to solve dilemma situations through value management”.

Similarly to von Groddeck (2011a, p. 74), we distance ourselves from Luhmann’s “pessimistic diagnoses” of values. In organisations, while values do not directly incite concrete action, they are reflected upon to formulate an identity that helps stabilise organisational social systems (von Groddeck, 2011a, 2011b). Organisational value systems can thus guide the organisational self-description, which in turn forms part of decision premises (Argandoña, 2003; Besio & Pronzini, 2014; Pruzan, 2001; Schnebel, 2000; Seidl, 2018). As such, organisational values become an integral part of the organisation’s autopoiesis. Following Besio and Pronzini (2014), values can be perceived as a moral compass within the organisation that emerges in the form of decision premises through the organisational system’s engagement with its system environment, and the respective values within said environment. We, therefore, do not engage with value communication and its function as such, but rather focus on the emergence of value systems as decision premises via the organisation’s self-description and identity.

The second tension concerns the system environment within which organisations operate and upon which they self-reflect. Luhmann (2012) functionally differentiates society into sub-systems, separating (inter alia) the economy from politics. Unsurprisingly, Luhmann’s theory is often perceived as at odds with Marxist conceptualisations of the political economy, as it seemingly rejects the very existence of such a unified field (Thornhill, 2013). Marx similarly observed the functional differentiation of the economy from politics taking place within capitalist society. However, from a Marxist perspective, this functional divorce of politics from the economy serves the depoliticisation of the economy that enables the reproduction of capitalist dominance. Yet, both Luhmann and Marx understand society as reproducing itself through “internal functional logic” (Thornhill, 2013, p. 272). Schecter (2017, 2019) argues that Marxist conceptualisations of the political economy and social systems theory can work together but requires detaching Luhmann’s theory from his own specific world-view.

To overcome these tensions in applying social systems theory to organisational values, we follow Harvie and Milburn (2010), who contend that one particular value system in society dominates over others. Specifically, they examine perceptions of value (singular), as manifested in the dominant value system, and the way it influences organisational behaviour. They point to the value system of the ‘global market’ as a case, where the dominant perception of value is market (or capitalist economic) value alone. This is reflected in various measures, such as Gross Domestic Product in the national accounts or market capitalisation in businesses that define the standard of value based on its quantitative relation to money as a universal equivalent. From this view the main purpose of businesses is to maximise profits, accumulate capital, and create economic growth (Banerjee et al., 2021). To this end, firms are expected to ‘innovate or die’, whereby continuous innovation driven by profit maximisation and economic growth is viewed as the pinnacle of economic and societal prosperity through the creation of new jobs, products, and services (Robra et al. 2023). However, these assumptions are neither natural nor given, they are the result of the dominant capitalist value system(s) in society which are generally regarded as natural and thus depoliticised.

The case could be made that the capitalist value system(s) are the values of the economic sub-system. However, capitalism needs to be understood as more than ‘the market’ or ‘the economy’. The economic system is intertwined with the political and cultural system, amongst others. Capitalism represents a social formation which dominates society’s structures, culture, and ideology in general. Despite this dominance other values or value systems within society can or indeed do exist. Yet, these values either exist in niches or are often subsumed under the dominance of the capitalist value system(s). For example, innovation is generally regarded as having to pay off financially and enable capital accumulation, rather than focusing on addressing societal needs first and foremost (Robra et al. 2023). Similarly, education has to be profitable, rather than teach knowledge and critical thinking. Further, education can be regarded as increasingly aligning its curriculum with capitalist values, helping to further enshrine capitalist values as the dominant ones (Parker, 2018; Ruuska, 2019). Therefore, to critically approach business ethics through organisational values, we need to examine how society’s values influence organisational values, considering that the *dominant* value system in society tends to align with capitalism, or indeed become the capitalist value system.

### Post-Growth Values as the Basis for an Alternative Value System

In a capitalist society, it is, arguably, easier for organisations such as businesses to align with the dominant capitalist values. This tension is intuitively recognised, but the inner workings require closer examination. From a social systems perspective, such an alignment allows for easier connections with the organisation’s system environment, hence reducing uncertainty of reproduction. It therefore makes autopoietic sense for organisations to align with the capitalist value system in a capitalist society. This means it is very likely (and understandable) for an organisation to seek to prioritise profits and growth, over its societal or environmental purpose, meaning that it will view its operations mainly on this economised level as well. This includes viewing morals, values, and ultimately ethics along the same economised lines (see Besio & Pronzini, 2014).

However, we can argue that the dominant values of society, and particularly its economic values, *condition* organisational values, but they do not necessarily *determine* them. Indeed, the system environmental relations of a social system do not translate to determinism (Luhmann, 1989, 2012). The existence of alternative organisations, as discussed further below, is good enough evidence that organisational values can emerge as alternative- and often in contention to “the singularity of capitalist production” (Gibson-Graham, 2002, p. 16). Harvie and Milburn (2010, p. 635) highlight this contentious emergence of organisational values as follows:

“[V]alue is also contested. [...] [T]his mode of organizing human activity—the capitalist mode of production—is not the only mode. Although the value system that is capitalism’s *sine qua non* values only market or economic value, it is not the only value system.”

Unsurprisingly, this reifies the breadth of alternative values and alternative value systems to the capitalist ones that can and do exist. Despite the dominance of the capitalist value system, alternative values can often be observed and examined in guiding organisational structures and behaviour. Alternative forms of organising attest to this, such as the extensively documented diverse economies cases, led by Gibson-Graham et al. (see Gibson-Graham, 2002, 2008) or the organisations of the Social and Solidarity Economy (SSE), which have been recognised by the United Nations^3^ or the International Labour Organization^4^ for their contribution to Sustainable Development Goals. This pluriverse of economic forms and practices, from social enterprises and cooperatives, to all forms of informal and hidden labour, do exist within the confines of—and often despite and against—capitalism, prioritising social and environmental values over profits. Which also means that organisations can and arguably do adopt alternative values to dominant capitalist imperatives, growth being at the core of them.

However, alternative organisational values prioritising sustainable and equitable practices are often contradictory to- and incompatible with values and an organisational culture related to economic growth (Dyck et al., 2019; Ikerd, 2024). These contradictions create economic trade-offs and various regulatory, financial, and institutional barriers that can limit the transformative potential of organisations aligned with social and ecological values, such as SSE organisations (Lionais, 2016; Oudeniotis & Tsobanoglou, 2020; Salustri, 2019). Contrary, post-growth organisations diverge from traditional growth-oriented models to better serve their intrinsic values (Cosme et al., 2017; Durand et al., 2024; Islar et al., 2024). Hence, our focus on post-growth serves to decipher how organisations may align with alternative values that are in direct contention with the dominant values in their system environment and the conditions defining their own viability and reproduction, as we further explain below.

Post-growth argues that the continued pursuit of economic growth is ecologically and socially undesirable. Hence, post-growth scholarship envisions a society without the need for economic growth and expansion (Schneider et al., 2010). To achieve sustainability, post-growth proposes an absolute reduction in both production and consumption (Hickel, 2020b). Post-growth’s aim for absolute reduction in economic activity can be interpreted to signify an unquestionable incompatibility with capitalism (Foster, 2011; Hickel, 2020a; van Griethuysen, 2010). Capitalism, here understood as a societal formation based on capital accumulation and valorisation which produces and requires a constant increase in economic activity and output, i.e. economic growth (see Foster et al., 2010). Parts of post-growth scholarship, particularly degrowth, have been presented as inherently anti-capitalist or post-capitalist (see Schmelzer et al., 2022). The adoption of post-growth organisational values, arguably, represents an alternative, contradiction, and opposition to the dominant value system(s) within capitalist society.

For instance, not-for-profit orientation or, at a minimum, an organisational purpose not based on profit-maximisation has been recognised as a key facet of organising economic activity from a post-growth perspective (Hinton, 2020, 2021; Wiefek & Heinitz, 2018). Likewise, legal structures such as cooperatives or social enterprises have been argued to be fitting in a post-growth context (Blauwhof, 2012; Johanisova et al., 2015; Nesterova, 2020). In the following, we briefly present four post-growth organisational values, synthesised from post-growth literature on organisations and beyond. These post-growth values contrast the dominant capitalist way of organising economic activity and its focus on profit maximisation through capital accumulation and valorisation. Viewed as singular, each of these values may also be found in a typical capitalist business. Hence, in the context of post-growth, the following four alternative organisational values should be considered as interconnected and strengthening each other.

### Democracy and Employee Well-Being

Within post-growth scholarship, democratic governance models and flat hierarchies (see e.g., Khmara & Kronenberg, 2018; Hinton, 2020, 2021) as well as a focus on employee well-being (see e.g., Nesterova, 2020; Hankammer et al., 2021) have been argued to be key facets for post-growth compatible organisations. Within capitalist business, employee well-being is generally only considered for retaining the workforce and ensuring productivity to ensure profitability. In contrast, from a post-growth perspective, the concern lies directly with the employee and their well-being. Both this and democratic governance are generally attributed to enabling an organisation to make choices that are in the employees’ and by extension the environment's interests, rather than focusing on profit maximisation and accumulation.

### Social Good and Eco-Conscious Purpose

Post-growth literature highlights organisational purposes focusing on social needs and environmental aspects (Froese et al., 2023; Hankammer et al., 2021; Hinton, 2020; Khmara & Kronenberg, 2018; Nesterova, 2020; Wiefek & Heinitz, 2018; Robra et al. 2023). This is generally connected to broad principles within post-growth scholarship emphasising social and ecological well-being (Kallis, 2018; Latouche, 2009; Parrique, 2020). Post-growth organisational values around social good and eco-consciousness first and foremost emphasise an organisational purpose of catering for societal needs and provisioning. This can again be seen in stark contrast to dominant capitalist values of profit-maximisation and capital accumulation, where social provisioning is if anything a by-product that is constantly undermined by the drive for-profit.

### Openness, Collaboration and Cooperation

The concept of sharing and collaborating in contrast to competition in broader terms is often emphasised in research on post-growth organisations (Froese et al., 2023; Hankammer et al., 2021; Wiefek & Heinitz, 2018). This is further connected to ideas of commoning (Helfrich & Bollier, 2015). Digital commons in particular are seen as a key facet for organisations to align with post-growth (see e.g., Hankammer & Kleer, 2018; Kostakis et al., 2018). This tentative connection is usually drawn through non-profit driven activities and creating as well as sharing knowledge and expertise openly without the prospects of financial gain (see Kostakis et al., 2018). Overall, this is in stark contrast to capitalist notions of secretly guarded knowledge in order to achieve competitive advantages to ultimately maximise profits for capital accumulation (Robra et al. 2023).

### Convivial Innovation and Technology

In business management, innovation and technology are generally seen as key business drivers to ensure profit maximisation and growth. Within post-growth scholarship, engagements with technology and innovation particularly emphasise Ivan Illich’s (2001) concept of conviviality. For example, Vetter’s (2018) conceptualisation of convivial technology can be interpreted to emphasise accessibility, appropriateness, adaptability, and a focus on ecologically as well as socially non-destructive practices. Post-growth compatible organisations ought to use, produce, and invent technology in a convivial manner. Robra et al. (2023) posit that post-growth innovation needs to focus on use-value rather than the purpose of exchange value for continuous capital valorisation and economic expansion. This means that post-growth aligned organisations need to invent and use technology in a socially as well as ecologically useful manner. Additionally, products need to be adaptable and repairable (Dietz & O’Neill, 2013; Kostakis et al., 2018). This is in stark contrast to the common capitalist practices, where planned obsolescence and non-adaptability have become the norm to further drive profits.

The tentative analytical framework represents a perspective to view organisational values as emerging through an organisation’s autopoiesis and its interaction with its system environment. Further, we acknowledge value(s) as a contested field in society and, thus, the coexistence of diverse and alternative value systems, albeit under dominance of capitalist values. This signifies a non-deterministic influence by the societal setting on the organisation that is, nevertheless, conditioned by capitalist economic prescriptions. From this departure point, we are able to investigate how post-growth organisational values might emerge in a more nuanced way. To this end, we investigate a French social cooperative with an explicit post-growth orientation among its members, and examine the emergence of its organisational values from a post-growth lens.

## Methods

We adopted a single-case study research approach (see Yin, 2003; Vincent & Wapshott, 2014) to be able to study an organisation and its organisational values in-depth. Further, our research design takes influence from participatory case study approaches (see Reilly, 2010), aiming at understanding the studied organisation and its contextual setting.^5^

Methodologically, social systems theory is exploratory and enables the analysis of how communication in organisations forms systemic structures (Besio & Pronzini, 2011; von Groddeck, 2011b). Through interviews and participant observation, researchers aim to understand decision-making processes and mechanisms, to infer decision communication and decision premises in an organisational system. In this context, the interviewee/participant (organisational member) is regarded as a first-order observer of the organisation, whereas the researcher is the second-order observer who observes how the organisational member observes their own organisation. At the second-order observation, the researcher interprets their observation through their own theoretical foundation and assumptions (von Groddeck, 2011b). Through this interpretation, the researcher becomes able to infer existing decision premises in an organisation.

The above in-depth approach can only be pursued in a single-case study research design, which allows for the kind of detailed and contextually rich insights that broader studies might not provide (Mariotto et al., 2014). Further, single-case studies offer the opportunity to triangulate multiple sources of information, such as in-depth interviews and participant observation (Wright et al., 2021), while a relatively small number of participants is allowing us to maintain research integrity in line with participatory research principles (see Reilly, 2010).

Following the above methodological notes, as well as social systems theory scholarship (see Besio & Pronzini, 2011), we collected data from a single-case study through in-depth interviews and participant observations during an interactive workshop. We conducted five semi-structured interviews with the then active five members of the organisation. As our research approached organisational values as decision premises, the interviewees were asked to describe and reflect upon the organisation, its purpose and values from the perspective of the organisation. Further questions focused on how the organisation’s purpose and values influenced actions in the organisation, with additional questions probing the perceived origin of said values and purpose. The interviews were conducted and recorded over online media, lasted from 40 to 90 min, and were transcribed for data analysis.

The interactive workshop was conducted in-person and after all interviews had been conducted. As with the interviews, the workshop participants were the then active five members of the organisation. Using an initial analysis of the interviews, the workshop firstly focused on the observed organisational values and purpose to triangulate these findings. For this, the participants were asked to, initially, reflect individually about the organisation's values and purpose. Afterwards, their notes were brought together to be reflected upon as a group (taking the perspective of the organisation), thus, creating a coherent picture of the organisation’s values. Additionally, the participants were asked to reflect on the sources of the organisational values, while explicitly asked to reflect on the organisation’s system environment and discuss the organisation’s place within it. As a group, the participants ordered their personal notes as influences in the organisation’s system environment, co-creating a value map, which was documented for analysis, alongside field notes collected throughout the workshop. Additionally, we presented the study’s preliminary findings to the participants on two occasions to further fine-tune the findings through participatory discussion and feedback.

Data analysis was divided into two stages. The first stage analysed the collected data (interview transcripts, workshop notes, and field notes) to ascertain the organisational values’ alignment with post-growth values. The analysis followed the described concept of a second-order observation, analysing the collected data to infer organisational values and then compare them against the four categories of post-growth values highlighted in Sect. “Post-Growth Values as the Basis for an Alternative Value System”. In the second stage, the collected data were analysed to understand the source and the emergence of the observed post-growth alignment. For this, the collected data was analysed in an interpretative way. Three themes directly emerged from this analysis, namely: (1) organisational history; (2) organisational members’ experience; and (3) observation of the system environment. We used these themes to code and interpret the data, as presented further below.

## Case Study

### Case Description

The French social cooperative *noesya* is established as a Société Coopérative de Production (SCOP). SCOP is a French legal governance structure that dictates rules regarding the organisation’s shareholders and profits. At least 51% of shareholders must be employees that hold at least 65% of the votes. Profits must be distributed between deposits in the organisation for the future (minimum 15%), the workers (minimum 25%) and the shareholders (must be below both worker and future deposit percentages). *Noesya* builds digital commons, develops low ecological impact websites, and creates interactive experiences. One of the organisation’s main projects is the free and open-source software *Osuny*. *Osuny* allows universities and research laboratories to create and operate high quality websites that support high accessibility standards, strong security protocols, low carbon emissions, and good retro-compatibility.^6^*Noesya* is looking to develop *Osuny* into a digital common where users will continue to develop it further for the wider community to use.

In the following subsections, we describe *noesya’s* organisational values (Sect. “Organisational Post-growth Values”) as well as their emergence (Sect. “The Source of* noesya’s* Post-Growth Values”). We interpret and discuss these findings, including structural implications in form of decision premises, in Sect. “Discussion”.

### Organisational Post-Growth Values

As stated in Sect. “Methods”, we compared the collected data against the theoretical post-growth values in Sect. “Post-Growth Values as the Basis for an Alternative Value System”, namely: (1) democracy and employee well-being; (2) social good eco-conscious purpose; (3) openness, collaboration and cooperation; and (4) convivial innovation and technology. In the following subsections, we therefore present our findings along these themes, highlighting *noesya’s* post-growth alignment.

#### Democracy and Employee Well-Being

All interviewees noted that democracy and employee/member well-being in the organisation is a key value of *noesya*. In this context, many interviewees emphasised equality and democratic decision-making as core aspects of the organisation. For example, one interviewee stated:

“We all are equal in *noesya*. That is why we chose to be a social cooperative, so that we all have the same say in decisions.”

This was further emphasised through several members bringing up the term “equality” to describe *noesya* in the workshop.

Some of the interviewees additionally described the organisation’s values of democracy in contrast to making money. One interviewee voiced:

“[I]t is not about the money. It is about democracy, we make decisions together.”

Similarly, when talking about the organisation’s focus on employee well-being, some interviews put this in contrast to the pursuit of profits. In this regard, one interviewee stated:

“We believe that [an organisation] is just the work tool of the employees, and that the well-being of the employees is more important than the profits of the shareholders”.

This was further reiterated in the workshop by all participants describing *noesya* as “people-centric instead of profits oriented”.

#### Social Good and Eco-Conscious Purpose

All interviewees emphasised that the websites *noesya* creates have to be useful for society as well as ecologically sustainable, for example:

“[W]e want to make great websites that are accessible for everyone, and with a low carbon footprint.”

“*Noesya* is committed to a quality, eco-designed, ethical and sustainable digital world.”

Some interviewees extended this social and ecological perspective to broader terms in relation to people and planet:

“[*Noesya* works] for the good of people and the planet. [...] So, we are trying to do some good in the digital world”.

“[*Noesya*] wants to create something that really helps people and is very useful.”

Within the workshop, the participants reiterated their organisation’s purpose through statements such as:

“Acting for the greater common good”.

“Respect people and environment”.

“Create sustainable website solutions”.

“Make the web greener and reduce its carbon impact”

“Help create better accessibility in the web”

As such, *noesya* clearly has a very eco-conscious and social good oriented purpose. All the interviewee’s put this organisational purpose into contrast with the broader industry of web-solutions. One interviewee stated the following:

“There is a lot of web-development that are interactive experiences with a lot of features, [usually not considering] the ecological footprint and the accessibility of the website. And so with *noesya* we are trying to show that you can create websites that are ecologically responsible and accessible.”

Additionally, to the contrast to the wider industry, some interviewees explicitly contrasted *noesya’s* social and ecological purpose again with the pursuit of profit maximisation, for example:

“The goal of [*noesya*] is not to make lots and lots of money, it’s to fix lots and lots of problems by doing the best craft we can.”

This contrasting was further highlighted in the workshop through statements such as:

“Build solutions for people instead of profits”

“Respond to needs, not creating ‘false’ needs”

As such, the organisational members further emphasised that *noesya’s* purpose stands in contrast to the dominant capitalist value of seeking to make profits.

#### Openness, Collaboration and Cooperation

All interviewees described open-source development or an open-source spirit at the heart of *noesya’s* activity. One interviewee described this in the following:

“Open-source is like a core value for *noesya* [...]. We want to create things that other people can use, so we also make them more accessible.”

Another interviewee emphasised practices to align with the open-source spirit:

“We try to document everything in our code, how we do things. [...] This is the base of the open-source spirit.”

Some interviewees further connected this to the concept of digital commons, for example:

“We create websites that are digital commons, so projects that are open-source and developed for the common good, like Osuny, [*noesya’s*] first digital common that we have created for [...] universities.”

In the workshop, the participants supported this focus on open-source and digital commons through statements like:

“Sharing practices in the creative commons”.

“Develop open-source technology”.

The majority of interviewees contrasted the organisation’s aim of creating digital commons with the pursuit of profits, for example:

“*Noesya’s* mission is to create digital commons, so that we can make big projects but do not focus on making a big profit from it. It is more focused on creating something with a great group of people. And creating something that is born from discussion and accessible to anyone.”

In this context, some interviewees further connected this to the contrast between competition and cooperation, for example:

“The first important point is going from a competitive world to a cooperative world, it’s shifting from this endless competition [...]. So, this is the biggest part, going from competitive to cooperative. Open-source is a way of doing that because we share what we do.”

This was also highlighted in the workshop through statements like:

“Turn markets (competition) into commons (cooperation)”.

Overall, *noesya* clearly values openness, collaboration, and cooperation. Further, the organisational members, again, saw the need to describe these values in contrast to profit-making, but also competition.

#### Convivial Innovation and Technology

While talking about the role innovation and technology play in the organisation’s work, all interviewees critically reflected on how technology was used in *noesya* as a tool to achieve the organisation’s purpose. One interviewee emphasised for example:

“We regard technology as a tool that we use for the commons and the common good.”

In the workshop, the participants supported this through statements like:

“[We] use technology to limit website carbon impact”

Many interviewees further emphasised that the organisation sought to counter obsolescence, for example:

“We aim to create robust and resilient solutions that fight against technological obsolescence.”

In the context of countering obsolescence, the interviewees further referred to retro-compatibility. For example, one interviewee stated:

“We have a responsibility as developers to create things that don’t have planned obsolescence. In this context, retro-compatibility is very important. We have a responsibility to develop things that are accessible from old devices. For me, this is the responsibility that we at *noesya* have, to keep things accessible.”

The workshop participants reiterated both the concepts of ‘retro-compatibility’ and ‘fighting obsolescence’ as key aspects in *noesya’s* work.

Many interviewees also mentioned maintenance as a key aspect of the work at *noesya*, for example:

“Maintenance is a big part of *noesya's* activity. As web technologies keep evolving, it's not enough to create a website and put it online. You have to ensure it keeps working overtime.”

“Maintenance is a very essential term, and it is very important for noesya, we are working long-term. Don’t try to fix it if it’s not broken, take care of something, so it can last as long as possible, that’s so simple.”

“We have a responsibility to maintain websites that can work with older devices as well.”

Within the workshop, the participants reiterated the focus on maintenance, retro-compatibility, and fighting obsolescence. While some interviewees put the focus on maintenance in contrast to creative destruction:

“Maintenance is very much against reboot, and new versions every time. It’s very anti Schumpeter [creative destruction] where you destroy and create new things, and it takes lots and lots of effort.”

Some interviewees also contrasted *noesya’s* use of technology and innovation with making a profit, for example:

“Usually tech is just used as a means to make money. The difference is that we don’t innovate in the sense of making a profit from it.”

“We are not trying to innovate to make more money. [...] But the key is that we are innovating to create useful stuff for people.”

Overall, *noesya’s* members emphasise using technology and innovation to do good and focusing on needs. The interviewees, again, felt the need to describe this in contrast to profit seeking.

### The Source of* noesya’s* Post-Growth Values

As described in Sect. “Methods”, three themes emerged from the analysis regarding the source of *noesya’s* organisational values: (1) organisational history; (2) organisational members’ experience; and (3) observation of the system environment. In the following, we present the findings along these themes.

#### Organisational History

When asked to reflect on the reason for *noesya’s* purpose, many interviewees started referring to the organisation’s legal structure as a social cooperative, for example:

“We see ourselves like more traditional cooperatives, so we really just want to do a good job and help people.”

Generally speaking, all interviewees emphasised that the organisation was a (social) cooperative when describing *noesya*, for example:

“*Noesya* is a cooperative, it is like a web-development studio but in a cooperative way.”

While referring to *noesya* as a SCOP, various interviewees further emphasised some of the legal implications of this. For example, one interviewee plainly stated:

“We have to invest most of our profits back into the company according to cooperative law. [...] The purpose of the cooperative is not to maximise profits.”

The organisation’s cooperative structure was further explained as being in place to protect the cooperative itself, for example:

“As a cooperative in France, we cannot be bought by another company. So every financial and administrative mechanism is for the good of the cooperative, it’s about protecting the cooperative.”

This protective mechanism was described as essential by all interviewees. In this context, they referred to a company previously founded and run by two of *noesya’s* members. Said company was bought out in a hostile takeover, as the below quote highlights:

“We built a previous company before, back in 2003, it was a very classical, limited company, so we were completely focused on the craft and not at all on the legal side of things and the governance. And the company was bought. And [two investors] bought us to use our craft solely for the purpose of using it as a means to make money. The way they made decisions showed that our craft was not important in their eyes.”

The interviewees described the organisation's cooperative structure as a direct result of this experience:

“We chose the social cooperative legal form to protect against venture capitalists and hostile takeovers.”

“The company structure we chose is not good if you want investors, because they would almost certainly never see their money again.”

While speaking about the choice of being a cooperative, some interviewees further contrasted the organisation’s legal structure with traditional capitalist firms, for example:

“We share a common rejection of the rules of traditional capitalistic companies, and a desire to do good work to improve the web of tomorrow. The traditional companies are mostly capitalist tools: you buy some shares, hoping that the value of the company will increase [...].”

Hence, the organisational members not only described *noesya’s* legal structure as a defence mechanism but also as a vehicle to contrast to capitalist norms.

#### Organisational Members’ Experience

When asked to explain the origin of *noesya’s* values, many interviewees started referring to their individual values and how they aligned with the organisational values. One interviewee for example stated:

“The organisation’s values are just the reflection of the personal values we share. [...] It’s not very difficult for me to follow the values of the organisation because we created *noesya* to reflect our own values.”

Another interviewee similarly voiced:

“We adopted these values naturally.”

When asked to elaborate on their personal values in connection to *noesya,* many interviewees started to describe working in more ‘traditional’ companies and realising that this did not align with their personal values. These interviewees described *noesya* and its values in contrast to these previous experiences, for example:

“I wanted to stop doing work just to make profits for other companies. [...] And I was not really comfortable with that. [...] And I was asking myself if I want to work for these companies where I do not align with their values around capitalism and to continuously sell, sell, sell. Instead, I want to create websites with real content and not only to continuously keep selling stuff or increase the number of clicks. So now *noesya* is like a redemption, where I do things that I am aligned with.”

Another interviewee reflected similarly on this:

“I was trying to reduce the gap between my beliefs, what I wanted to do and what I really did. Now, [working for *noesya*], the gap is reduced.”

In other words, *noesya* became a place for the interviewees where their personal values were no longer in contrast to the organisational values.

#### Observation of System Environment

When asked to reflect on how the participants viewed their organisation in the context of society, the participants started to contrast their organisation’s activity with the pursuit of profits (similarly as described in Sect. “Organisational Post-growth Values”). The participants emphasised this through statements such as:

“Work to maximise profits is the norm. *Noesya* works for fair profit.”

“The norm is that money stands above all. In *noesya* we want usefulness above all.”

Similarly, the workshop participants described *noesya*’s perspective on innovation and technology in contrast to perceived norms, like:

“Outside is a tech race. In *noesya* we focus on better tech.”

“The norm is to always use the latest tech tools. We use the best tech tools for the job.”

The participants in this context also referred to other more ‘traditional’ web-development organisations, the participants explained that they used these as anti-examples (i.e. examples of how not to be) for *noesya*. When asked to reflect on these contrasts, one participant described the organisation in the following:

“We are a sane island in an ocean of madness.”

The other participants agreed with this description but also voiced that the organisation is not alone, or indeed the only ‘sane island’. This was highlighted through descriptions such as:

“*Noesya* is in a small circle of companies focused on green and eco solutions.”

This was similarly described in by some interviewees, for example:

“There are some organisations that work on how to make the web greener, so this is a clear influence for us. We are not the first organisation that tries to make the internet greener, so we engage with some of these companies.”

In the interviews, the organisation’s members also reflected upon how the organisation engaged with its surroundings, i.e. its system environment. Many interviews emphasised that their values influenced how they engaged with potential clients, for example:

“We have stopped working with people that are rejecting our values. We use our values as a natural filter.”

Some interviewees however also emphasised that:

“We also have to maintain some of our previous projects, which might not align with our values anymore. [...] But this is also connected to our values, as we believe in maintenance instead of always creating new solutions.”

Hence, even though the organisation has to maintain projects that might not align with the organisation’s value at first glance, the organisational members connected this back to the organisational values emphasising the need for maintenance.

## Discussion

The values described in Sect. “Organisational Post-growth Values” can be regarded as *noesya’s* values. We can infer this as the interviewees/participants at the first-order observation describe them as such. Further, the interviewees described at several points how the values they described guided and influenced their actions in the name of the organisation. Together, these values represent the organisational value system that determines what behaviour is deemed ethically correct. Hence, regardless of their locus, *noesya’s* organisational values can be considered part of the organisation’s decision premises, thereby its structures, and ultimately influencing its autopoiesis (Luhmann, 2018; Seidl, 2018). As the interviewees did not refer to specific processes or mission statements but rather to ‘the way things are done’, *noesya*’s organisational values seemingly influence the organisation’s modus operandi in general through unwritten rules rather than tangible structures. That being said, the organisation’s legal structure (i.e. SCOP) seems to enable, support, and reinforce the organisational values and vice versa.

In regard to post-growth, the observed organisational values align with the a priori description of post-growth values (see Sect. “Post-Growth Values as the Basis for an Alternative Value System”). Our findings, therefore, show that an organisation can adopt post-growth organisational values and carve out an alternative ethical framework. This alternative ethical framework has been largely brought forth in contrast to the dominant values of the organisation’s system environment, which is predominantly defined by the capitalist value system. This is underlined by the interviewees/participants proactively describing *noesya* in contrast to how a business is dominantly perceived to operate in line with an endless pursuit of profit. Hence, similar to post-growth values, *noesya’s* values can be regarded as contrasting the dominant capitalist values and value system(s).

In the following sections, we elaborate on these findings in relation to the sources and influence of *noesya’s* values. We further discuss our findings in relation to business ethics literature, as well as their significance for a potential post-growth transformation.

### The “Other Than Capital”

Von Groddeck (2011a) highlights how interviewees describe an organisation’s identity and values through explicitly referring to an ‘outside’—in other words, the organisation’s system environment. As a social system has to draw a distinction from its system environment, the uniqueness of the organisation’s values and identity can only be described in contrast to what the organisation perceives to be in its system environment (von Groddeck, 2011a). Following this interpretation, it is not surprising that *noesya’s* members described their organisation and its values in contrast to the organisation’s system environment. Post-growth values in *noesya* seem to emerge as an opportunity for the organisation to draw a distinction from the capitalist value system, reflecting on itself as something “other than capital” (De Angelis, 2007, p. 13). The key question regarding the emergence of *noesya’s* alternative value system is therefore not why the organisation contrasts its values, but rather why it contrasts them to the dominant capitalist value system(s). To understand the emergence of post-growth organisational values in *noesya* it is vital to discuss how the organisation interprets its system environment and, hence, its need to contrast the organisation against capitalism. For this, we need to draw connections to the three themes described in Sect. “The Source of* noesya’s* Post-Growth Values” related to the source of *noesya*’s values, namely, (1) organisational history; (2) organisational members’ experience; and (3) observation of the system environment.

According to Schnebel (2000) organisational values are generally rooted in the organisation’s traditions, including organisational history (see also Hofstede, 1980). *Noesya’s* history is relatively short due to the organisation’s relative infancy. However, many interviewees referred to a previous business of two of the founders, which was bought in a hostile takeover, when explaining the source of *noesya’s* values. Hence, we can infer that the history from a previous organisation influences *noesya*’s organisational value system.

Similarly, many interviewees described their own personal experiences in previous employment and how their personal values misaligned, while feeling more at home within *noesya* and its values. Many scholars (see e.g., Wiener, 1988; Agle & Caldwell, 1999; Padaki, 2000; Pruzan, 2001) have argued that an organisation’s members are a source of organisational values. Hence, we can infer that *noesya’s* members’ personal experience and reflection play a part in the emergence of the organisation’s values.

When describing both, their personal experience and the organisational history, the organisational members referred to capitalism as the norm while describing capitalist characteristics such as profit maximisation, planned obsolescence, and competition as highly problematic for society and the environment. This represents a particular world-view, or in other words, a particular interpretation of the organisation’s system environment. The experience the organisational members made as well as the history of the previous business seem to have acted as a trigger point to help the emergence of reflections on the general societal setting. In other words, the organisational members started to reflect on the dominant capitalist values in its system environment and an organisation’s positionality in said setting.

This reflection in turn led *noesya*’s members to choose a particular legal structure to contrast the organisation to the dominant norms. All interviewees saw the need to explicitly describe *noesya* as a social cooperative. This is significant, as Seidl (2018) makes the case that an organisation’s self-description plays a key role for organisational identity and thus the organisation’s autopoiesis and modus operandi. An organisation’s self-description can even be regarded as part of its decision premises (Seidl, 2018). The choice of SCOP as an organisational structure has clear legal implications, as also described by the interviewees. Further, the social cooperative legal structure can be regarded as accommodating an alternative value system, as it does not allow for the pursuit of profit maximisation, to which the alternative values stand in contrast. Hence, the self-description as a social cooperative is further used as a basis for defining the organisation’s activities, values, and decisions in line with this description. This was emphasised by all interviewees using the self-description as a social cooperative to explain and define the organisation’s purpose, values, and activities.

**Fig. 1:**
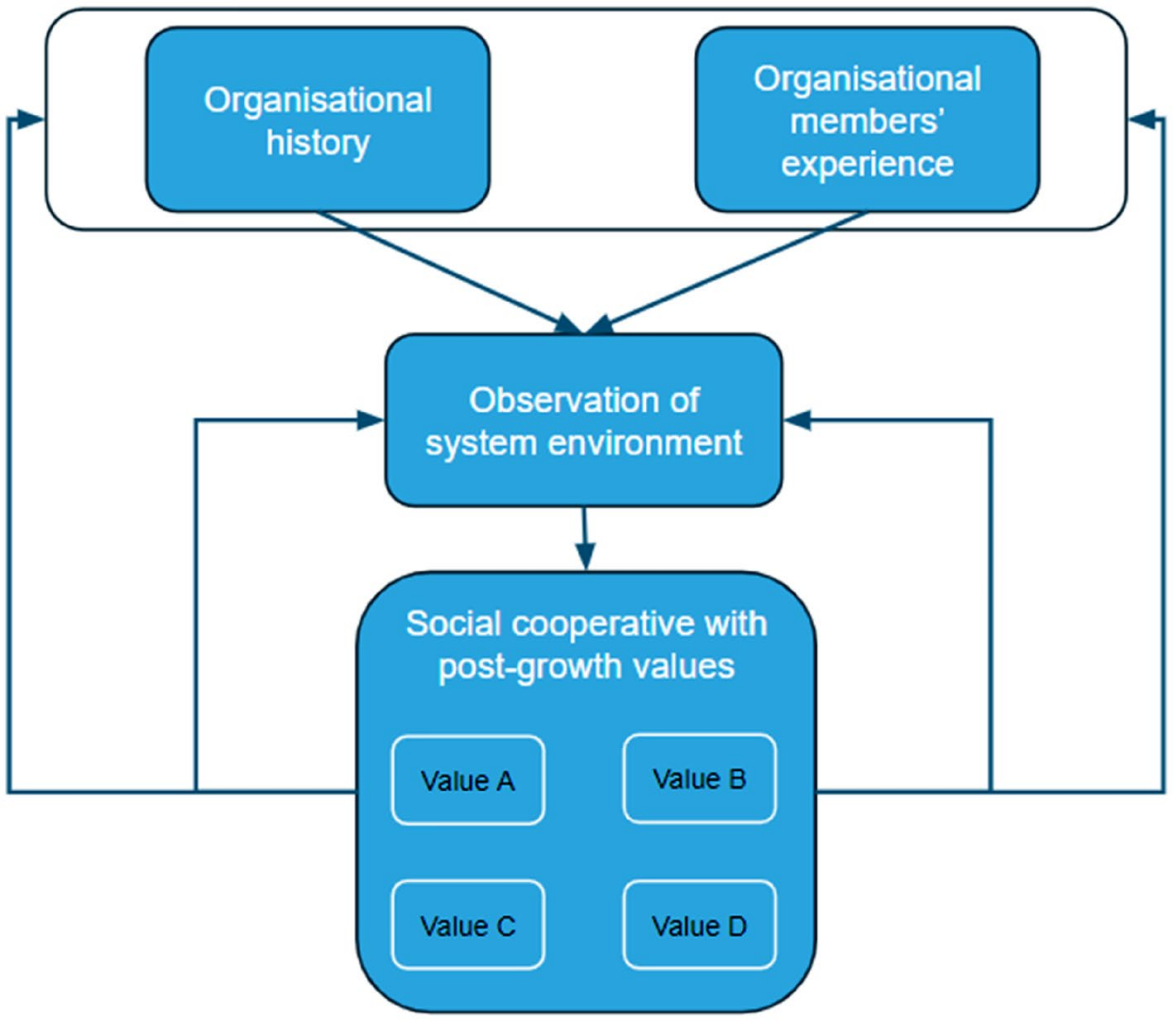
Emergence of organisational values in *noesya*

In summary, *noesya*’s post-growth organisational values can be understood as an outcome of its members’ perception of the system environment, informed by their experience and organisational history, which further results in the perceived need for particular legal structures and values that are in contrast to the dominant capitalist values. According to Pruzan (2001, p. 278) “an organization is […] considered to be a social system with the self-referential ability to describe itself and reflect upon itself on the basis of its shared values”. Hence, the case can be made that by imbuing the organisation with their values, *noesya*’s members are also imbuing the organisation with their own personal experience as well as a particular interpretation of the organisation’s history, and way to observe its system environment. Additionally, the organisational values can be seen as the basis to reflect on and reinterpret the members’ experience and the organisation’s history, as well as the way to observe the organisation’s system environment. As such, the organisational values are simultaneously a product of the organisation’s history and the members’ experiences, and the basis to reflect upon them. These connections are illustrated in Fig. 1 below.

### Value Struggles in Business Ethics

Our findings highlight a contrast between on the one hand ecological and sustainability values, and on the other hand the values of the economic environment within which businesses operate. Analysing today’s business terrain as the main system environment of economic organisations (Besio & Pronzini, 2014; Luhmann, 2018), we can observe the dominance of capitalist value systems around the purpose and processes of the economy and markets (see Harvie & Milburn, 2010). Within these dominant value systems, sustainability practices will likely only be adopted if they pay off in a *capitalist* economic sense (Joyner & Payne, 2002; Porter & Kramer, 2006). In this context, the predominantly moral call for businesses and corporations to become more sustainable is alone insufficient (Besio & Pronzini, 2014). The ethical frameworks commonly adopted by organisations operate using values that include, at best, reformist sustainability concepts that do not harm profit-making.

Moreover, the calls for an integrated ethical theory and practice of the firm (e.g., see Yazdani & Murad, 2015) may contribute to changes in how organisation and management scholars engage with business conduct but arguably offer little in addressing the prominence of economic performance motives over ethical conduct in actual business practice. For instance, when an organisation explicitly aims to align with post-growth values, as is the case with *noesya*, this alignment does not occur harmoniously. Businesses are generally expected to align with the capitalist value systems, at the core of which is the spirit of a rational and systematic pursuit of profit (see Weber, 2016). The emphasis here is on ‘rational’ and ‘systematic’, which elevate the pursuit of profit from a mere business function to a value system that permeates all layers of how the economy functions. From a social systems view of organisations we can understand how a firm rationalising profits over virtue creates less friction between the organisational value system and the dominant ones, thus, making its reproduction far more likely.

Yet, as mentioned before, this alignment is non-deterministic and values can be contested (see Harvie & Milburn, 2010). Our findings reaffirm such potential, while illustrating why and how post-growth organisational values emerge in contrast to the capitalist ones. To understand how ethical motivations may be elevated over market imperatives in organisations it is, arguably, important to engage with both value (singular) and values (plural) as a field of contest and struggle. That is, discussion on organisational values, which define what is important for the organisation, needs to also reflect on how these values translate in the perception of value, which defines how (business) conduct acquires meaning in the broader social whole (Graeber, 2001). Ethical considerations in business conduct are undoubtedly important and often influence organisational behaviour, however, they rarely influence how value is perceived in the economy. Ultimately, the value of any product, employee, or the organisation as a whole is always translated, as far as the organisation’s reproduction is concerned, in some sort of abstract monetary equivalence, be it revenue, remuneration, rewards, or its bottom line. These perceptions of value, in turn, influence organising as a practice, be it in businesses, non-profits, or governments, whereby what is valued is not any material or immaterial wealth co-created but the process of continuous competition driven by the rationality of abstract valuation (Harvie & Milburn, 2010).

Alternative organisations, such as cooperatives and social enterprises, or alternative forms of organising, including informal provisioning networks and communities, provide a favourable field to investigate the emergence of alternative and/or post-growth organisational values that contrast the dominant perception of value. Such cases exhibit diverse organisational practices, driven by non-capitalist, anti-capitalist, or more-than-capitalist ethics (see Gibson-Graham, 2002, 2008) that directly contrast the conventional canons of employment, management, and distribution through which value is co-created in economy and society. A pluriverse of economic forms exists within the confines of—and often despite and against—capitalism, acknowledging economic organising as a space for ethical action, which can be shaped to serve the well-being of the people and planet (Gibson-Graham et al., 2013).

Research on the contribution of organisational forms, such as SSE organisations mentioned further above, to sustainability abounds. Some key findings focus on aspects related to the localisation of consumption and supply against global market practices (Gea Wijers, 2019); the fostering of inclusive employment; and the reduction of inequalities, enhancing accountability and responsible business conduct (Díaz De León et al., 2021; Filippi et al., 2023). Further studies (e.g., Rincon-Roldan & Lopez-Cabrales, 2022) draw direct links between values of support, respect, and responsibility, typically found in SSE organisations, and sustainable management and organising, promoting practices that enhance ability, motivation, and opportunity. Energy cooperatives have been linked to more sustainable energy use (Bauwens & Eyre, 2017), offering solutions for energy and environmental issues, while stabilising local economies through fair distribution systems, mobilising social capital to foster stronger community relations, crucial for energy transition (Bauwens & Defourny, 2017), and contributing to the overall common good and sustainability (Besio et al., 2022). Elsewhere, energy communities have been examined as niche initiatives with a degrowth transformative capacity (Vrettos et al., 2024).

In relation to *noesya*, further above we discussed the importance of the self-description of a social cooperative that has been emphasised in the interviews. Through this lens, the organisation’s members contrast their alternative values against those of their system environment, as they lean on a diverse set of values and practices formulating an alternative organisational ethics. SSE organisations vary across different contexts and settings, but are coalesced around a common set of principles that define their purpose and conduct and formulate the basis for the emergence of relevant organisational values. For instance, cooperatives have been illustrated for their capacity to shape individual identities around shared values, such as reciprocity, equality and cooperation (Reedy et al., 2016), which are often directly juxtaposed against global capitalism (Cheney et al., 2014). They, thus, embody and reproduce alternative moral values that arguably prefigure an alternative economy (such as a post-growth economy) altogether (Schiller-Merkens, 2024).

The connection, implicit or explicit, of all the above-described transformative aspects to alternative values that guide organisational behaviour is apparent, yet has not been systematically engaged with. We build upon this rich and diversified body of inquiry and contribute to it with an in-depth investigation on alternative value systems, attempting to decipher the interplay between alternative values, such as the ones manifested and embodied in SSE organisations, and the dominant capitalist value system. This would allow business ethics scholarship and practice to better delve into the constraints and contradictions of organising around alternative values that place sustainability and societal equality over profits, and evaluate how ethical conduct may be integrated into organisational theory and practice. Despite the stark divergences in structures and needs for their reproduction found in different organisational forms, there are, arguably, useful lessons to be drawn in unveiling the role of ethics in alternative, and specifically post-growth, economic organisation and the interpretation of ethical considerations into organisational practice.

### Organising for a Post-Growth Transformation?

Similarly to Wiefek and Heinitz (2018), our findings reaffirm that organisations might adopt post-growth values, while we also add that these values can be the basis for an alternative value system. Additionally, our findings highlight that in our case, post-growth values emerge through actively contrasting them to the capitalist value system. Hence, for post-growth values to emerge, it is vital that individual values of organisational members align (see Nesterova, 2021). But crucially, this also needs to combine with a view of the organisation’s system environment critical of capitalism. This holds significance for post-growth scholarship. Post-growth can generally be regarded as entailing overcoming capitalism, its mode of production and wider political economy (Akbulut, 2021; Löwy et al., 2022; Schmelzer et al., 2022). Research on organisations in the context of post-growth often lacks an acknowledgment of capitalism and its political economy or indeed that post-growth entails overcoming these structures (Robra & Hinton 2024).

Within post-growth scholarship, the role of organisation in helping a post-growth society emerge is generally unclear or not discussed. In the context of organisational values, Wiefek and Heinitz (2018, p. 329) conclude that “further research is necessary to explore the extent to which the companies’ compliance with Latouche’s eight ‘R’s [i.e. post-growth values] is indeed contributing to a societal transition towards degrowth”. In this direction, as our findings highlight that post-growth organisational values emerge in contrast and critique to dominant capitalist values, we believe to be able to point into one of several fruitful directions.

*Noesya*’s post-growth organisational values are connected to a system environment view that is highly critical of capitalism. In other words, the organisation has started to critique, oppose, and ethically question the dominant political and economic values in its system environment. Robra and Nesterova (2023) make the case that political critique of capitalism is a key facet that thus far has received little to no attention within the scholarship on post-growth organisations. Robra et al. (2021) argue that political post-growth alignment that simultaneously aims to scale-wide across organisations and societal structures is needed. An important question for post-growth scholarship is therefore how critical observation of capitalism, as found in *noesya*, can help a scaling-wide of post-growth values to challenge the dominant capitalist values, ethics, and structures. Hence, future research needs to set a focus on how organisational networks might help post-growth organisational values to scale-wide.

## Concluding Remarks

In this paper, we operationalised ethics in organisations through their value systems. We set out to better understand how post-growth organisational values might emerge in a society where capitalism dominates. By employing a tentative theoretical framework that borrowed and synthesised from Luhmann’s social systems theory and Marxist interpretations of political economy, we explored the emergence of post-growth organisational values in contrast to an organisational system environment mainly dominated by capitalist values. Our engagement with the social cooperative *noesya* reaffirms that post-growth values, which stand in stark contrast to the dominant capitalist value systems, can emerge in organisations and formulate their ethical basis. Further, this means that economic organisations can operate using an alternative ethical framework not subsumed under the capitalist value system. On the other hand, our findings highlight how post-growth organisational values emerged through a particular way of observing the organisation’s system environment, influenced by the organisation’s history and the organisational members’ experiences. In future research it is vital to understand how organisational self-reflection can further influence post-growth alignment through scaling-wide, such as in networks of like-minded organisations, thus fostering wider societal post-growth transformations.

## Footnotes

1 For readability, we refer to both degrowth and post-growth when we mention ‘post-growth’ in this article. We are very much aware of the intricate and different nuances between the two concepts. However, in this article, we believe it is more important to be able to refer to both degrowth and post-growth literature as one.

2 By ‘economic organisation’ we refer to business organisations, such as corporations, firms, and alternative forms of business, such as cooperatives alike.

3 Resolution adopted by the General Assembly on 18 April 2023. Available at: https://documents.un.org/doc/undoc/gen/n23/118/68/pdf/n2311868.pdf.

4 ILC.110/Resolution II. Available at: https://www.ilo.org/resource/ilc/110/resolution-concerning-decent-work-and-social-and-solidarity-economy.

5 Regarding the participatory aspect of the study, the third author of the paper is a co-founder of the studied organisation. To counteract any potential conflict or bias, this author was solely involved in the analysis and discussion phases of the research process. This allowed a deeper understanding of the collected data by introducing insider reflection on the findings and their meaning.

6 Retro-compatibility, referring here to older digital devices such as PCs and mobile phones continuing to be compatible with newer software iterations. Hence, countering the need to upgrade the hardware.
